# Spatial pattern and influential factors for early marriage: evidence from Bangladesh Demographic Health Survey 2017-18 data

**DOI:** 10.1186/s12905-023-02469-y

**Published:** 2023-06-20

**Authors:** Md Arif Billah, Md. Mostaured Ali Khan, Syed Manzoor Ahmed Hanifi, M. Mofizul Islam, Md. Nuruzzaman Khan

**Affiliations:** 1 Health System and Population Studies Division, International Centre for Diarrhoeal Disease Research, Bangladesh (icddr,b), 68 Shaheed Tajuddin Ahmed Sarani, Mohakhali, Dhaka, 1212 Bangladesh; 2 Maternal and Child Health Division, International Centre for Diarrhoeal Disease Research, Bangladesh (icddr,b), 68 Shaheed Tajuddin Ahmed Sarani, Mohakhali, Dhaka, 1212 Bangladesh; 3 Department of Public Health, School of Psychology and Public Health, La Trobe University, Bundoora, VIC 3086 Australia; 4 Department of Population Science, Jatiya Kabi Kazi Nazrul Islam University, Namapara, Mymensingh, 2220 Bangladesh

**Keywords:** Early marriage, Spatial analysis, Influential factors, Multi-level modelling, Bangladesh

## Abstract

**Background:**

Early marriage is highly prevalent in Bangladesh. It is linked with a range of adverse outcomes, including maternal and child mortality. However, research on regional variations and factors associated with early marriage is scarce in Bangladesh. This study aimed to explore the geographical variations and predictors of early marriage in Bangladesh.

**Methods:**

Data of women aged 20–24 in the Bangladesh Demographic and Health Survey 2017–18 were analysed. The occurrence of early marriage was the outcome variable. Explanatory variables were several individual-, household- and community-level factors. Geographical hot spots and cold spots of early marriage were first determined using Global Moran’s I statistic. Multilevel mixed-effect Poisson regression was used to determine the association of early marriage with individual-, household-, and community-level factors.

**Results:**

Almost 59% of women aged 20–24 reported they were married before reached 18. The hotspots of early marriage were mainly concentrated in Rajshahi, Rangpur and Barishal, and the cold spots were in Sylhet and Chattogram divisions. The prevalence of early marriage was lower among higher educated (adjusted prevalence ratio (aPR): 0.45; 95% CI: 0.40, 0.52), and non-Muslim women (aPR: 0.89; 95% CI: 0.79, 0.99) than their counterparts. Higher community-level poverty was significantly associated with early marriage (aPR, 1.16; 95% CI: 1.04, 1.29).

**Conclusion:**

The study concludes that promoting girls’ education, awareness-building programs about the adverse effects of early marriage and proper application of the child marriage restraint act, particularly in disadvantaged communities are recommended.

## Introduction

Early marriage – marriage before the age of 18 years – is a form of sexual violence that is highly prevalent in low- and middle-income countries (LMICs), including Bangladesh [1]. Early marriage violates sexual and reproductive health and rights, although often neglected in LMICs [1–7]. Approximately 37% of marriages in LMICs occur at an early age, of which 28% are in South Asian countries [2, 5]. The rate is even higher in Bangladesh, where around 51% of all first marriages occur before girls reach 18 [8]. This high prevalence ranked the country 8^th^ in the world and 1^st^ among South Asian countries in terms of the rates of child marriage [9]. The rate has dropped over the years, albeit slightly and not as much as one would expect from the progress that Bangladesh has made in other sectors, including increasing girls’ enrolment in education and improving the status of women in society [10].

Marriage before age 18, the transition period from childhood to adulthood, could have consequences on women’s well-being [11]. This includes but is not limited to drop-out from education and occupation, intimate partner violence, hostile marital relationships and increased divorce and separation rates [12, 13]. Moreover, around half of the women who experience early marriage get their first pregnancy before they reach age 20 [9]. Childbearing may be risky for maternal and child health, as women at this stage may not be physically ready for conception and delivery [14, 15]. Pregnancy complications, preterm birth, low birth weight, stillbirths and maternal and child mortality are some of the common adverse outcomes of early marriage [16–20]. Along with physical immaturity, higher malnutrition, lower use of maternal healthcare services, risky fertility behaviour and unwanted pregnancy are also highly prevalent among early-aged married women [21–24]. These are also associated with increased occurrences of child morbidity and mortality. Early marriage, therefore, presents a challenge to Bangladesh and other LMICs to achieve the Sustainable Development Goals (SDGs) 3, health and well-being for all, by 2030 [25, 26]. Bangladesh aims to drastically reduce early marriage by 2041 [8, 9, 27]. To achieve this goal, Bangladesh needs evidence-based policies and programs. Understanding multidimensional factors associated with early marriage is critical for that.

However, studies thus far conducted in LMICs primarily focused on individual-level factors. These studies indicate that early marriage is more common among women from low socioeconomic tiers, with low levels of education [16, 28–38] and/or from conservative families [36, 39–45]. Besides, existing evidence suggests a significant role of community-level factors such as cultural and societal norms in early marriage [7, 14, 32, 34, 37, 38, 41, 43, 44, 46–49]. These predictors vary across regions in most LMICs, including Bangladesh [10, 33, 45, 50] and the strengths of associations are usually stronger for rural than urban women [37, 41, 51, 52]. The possible reasons for this variation are different cultural and social norms, education- and poverty-level across the communities [42, 48, 53] and indicates a hierarchical effect of the individual- and household-and community-level factors. However, to our knowledge, the association between early marriage occurrence and community-level predictors has not yet been explored in Bangladesh and most other LMICs, although a study presented regional-level estimates and likelihood of early marriage [36]. However, estimates of larger regions often fail to represent the distinctiveness of early marriage in the smallest geographical spaces. Therefore, this study aimed to determine the hot spots and cold spots of early marriage and the predictors of early marriage in Bangladesh.

## Methods

### Data source and study population

We analysed nationally representative Bangladesh Demographic Health Survey 2017-18 data. The National Institute of Population Research and Training (NIPORT) conducted this survey as part of the Demographic and Health Survey (DHS) program. The Ministry of Health and Family Welfare of Bangladesh supervised the data collection procedure. The target population for this survey was women of reproductive age (15–49 years) of the selected households. The households were selected using a two-stage stratified random sampling procedure. In the first stage, 675 primary sampling units (PSUs) were selected randomly from the list of 293,579 PSUs generated by the Bangladesh Bureau of Statistics (BBS) as part of the 2011 national census. Data was collected from 672 of these PSUs and the remaining three PSUs were excluded due to flooding. Geographical locations were also recorded using Global Positioning System (GPS). In the second phase, 30 households were randomly selected from each of the selected PSUs. This resulted in a list of 20,160 households, of which 19,457 households were interviewed. A total of 20,127 ever-married women between 15 and 49 years of age were interviewed based on the inclusion criteria of the BDHS survey, with a non-response rate of < 1%. The selected respondents were interviewed on reproductive health and maternal and child health indicators. Details of the sampling strategy have been published elsewhere [54].

### Sample

Data collected from 4,155 women who met the inclusion criteria were analysed in this study. The inclusion criteria were: (i) women aged 20–24 at the time of the survey and (ii) reported their marital status. We included only 20–24 years aged women to reduce the recall bias in reporting marital age. Another important reason for including only 20–24 years aged women was to ensure the generated estimates are relatively current because the older aged group may have married many years back when the country’s socio-demographic conditions were substantially different. BDHS only included ever-married women, although it provided an adjustment factor (all women factor) to consider the unmarried women in the analysis. We adjusted our analysis for that factor by following the DHS guideline [54].

### Outcome variable

Women’s age at marriage (with the first spouse in case women reported multiple marriages) was our outcome of interest classified as early marriage (occurred before 18 years) and not early marriage (occurred at 18 years or later). During the survey, women were asked the following question: “How old were you when you first started living with your husband?”. The response recorded was classified as early marriage and not early marriage.

### Explanatory variables

Several individual-, household-, and community-level factors were considered explanatory variables. The variables were selected in two stages. In the first stage, using several databases, we conducted a comprehensive search for the relevant papers published in LMICs, particularly in Bangladesh [7, 10, 29, 31, 37, 38, 41, 43–45, 55–57]. The factors that were found to be significantly associated with early marriage in the identified papers were then sorted and their availability was checked in the BDHS data. Finally, the variables that were found available in our data were considered for analysis and classified as individual-, household- and community-level factors as per the socio-ecological model of health [58–62]. Individual-level factors were women’s education (no formal education, primary, secondary and higher), husbands’ education (same categories as women’s education) and mass media exposure (no, yes). Household-level factors were number of family members (small (≤ 4 members) and large (> 4 members)), religion (Muslim, on-Muslim) and wealth index (poorer, poor, middle, rich, richer). The factors that were considered at the community level were the place of residence (urban and rural) and region of residence (Barishal, Chattogram, Dhaka, Khulna, Mymensingh, Rajshahi, Sylhet). In addition, we also examined community-level variables, namely community-level illiteracy and community-level poverty. Since these variables were not directly available in the survey data, we employed a two-step approach to measure them. Firstly, we calculated the proportions of uneducated women and women residing in resource-poor (i.e., women of poorer and poorest wealth quintiles) households at the cluster level. These proportions served as proxies for community-level illiteracy and community-level poverty, respectively. Subsequently, we categorized these cluster level proportions into three distinct categories: low (< 25%), moderate (25–50%), and high (> 50%) for community-level illiteracy; and low (≤ 15%), moderate (1640%), and high (> 40%) for community-level poverty. The detailed calculation procedure for deriving these variables has been previously published [63, 64], and followed that established approach to ensure consistency and comparability.

### Statistical analysis

Descriptive statistics – frequency and percentage distribution – were used to describe the characteristics of the respondents. Distribution of early marriage across individual-, household-, and community-level factors was also presented. Utilizing Global Moran’s I statistic, we conducted an analyses to identify regions characterized by relatively high and low prevalence of early marriage, which refer to as “hot spots” and “cold spots”, respectively. This statistical measure allowed us to assess the spatial clustering of early marriage rates.

To computed the Global Moran’s I statistic, we first calculated the mean and variance for the dependent variable, namely early marriage prevalence. Then, we determine the deviation of each feature value from the mean by corresponding subtraction. Subsequently, we multiplied the eviations of all neighbouring features together, resulting in a cross-product ranging from +1 to -1. Deviations for all neighbouring features were then multiplied together to create a cross-product that ranging from +1 to -1. In the context of Moran’s I, a value of +1 indicates strong positive spatial clustering, meaning high values tend to cluster near other high values, and low values cluster near other low values. Conversely, a value of -1 suggests repulsion, where high values repel other high values and tend to be near low values. When the cross-product values balance negative cross-product values, Moran’s I tends to approach 0 [65, 66].

Multilevel mixed-effect Poisson regression with robust variance was used to explore the predictors of early marriage. In BDHS, individuals were nested in households and households were nested within clusters. This hierarchical structure of the data creates dependency among the respondents’ responses, e.g., individual from same household or cluster provided a similar response from a different household or cluster. Moreover, the prevalence of early marriage, the outcome variable, was around 60%. Ordinary logistic regressions do not produce precise results for hierarchical data or when the prevalence of outcome variable is > 10%. Multilevel mixed-effect Poisson regressions with robust variance can address both issues [67, 68]. We developed five models, where Model 1 was the null model used to explore the cluster-level variation of early marriage in Bangladesh. Individual- and household-level factors were considered in models 2 and 3, respectively. Community-level factors were considered in Model 4. Model 5 was the final model that included all individual-, household- and community-level factors. We reported intra-class correlation (ICC), Akaike Information Criteria (AIC) and Bayesian Information Criteria (BIC) for all models. Results are reported as Prevalence Ratio (PR) with its 95% Confidence Interval (95% CI). All analyses were conducted using the statistical software STATA version 15.1 (Release 15. College Station, TX: Stata Corp LLC.).

## Results

### Characteristics of the study sample

Of the 4,155 women of age 20–24 years whose data we analysed, approximately 46% completed secondary education (Table 1). Around 38% of the husbands of the participants also completed secondary education. About 16% of women were from households of the poorest wealth quintile. Over 57% of women were from large families. Around two-thirds lived in rural areas and 27% were from the Dhaka division. Around 57% resided in the community with moderate poverty.

**Table 1:** Percentage distribution of early marriage by different socioeconomic characteristics of the sample

**Characteristics**	**Total respondents,** **N (%)**^**g**^	**Early marriage**^**h**^ **n (%)**
**Prevalence of early marriage**		**58.85 (57.21–60.47)**
**Women’s age (in years)**^**a**^		
20	876 (21.08)	482 (55.01)
21	867 (20.85)	527 (60.76)
22	858 (20.65)	494 (57.54)
23	746 (17.94)	454 (60.89)
24	809 (19.47)	489 (60.47)
**Women’s education**^**b**^		
No formal education	145 (3.49)	109 (75.03)
Primary	908 (21.84)	666 (73.32)
Secondary	1905 (45.78)	1368 (71.80)
Higher	1202 (28.90)	303 (25.20)
**Husband’s education**^**a, f**^		
No formal education	423 (10.53)	305 (72.16)
Primary	1289 (32.09)	844 (65.49)
Secondary	1527 (38.02)	877 (57.42)
Higher	777 (19.35)	327 (42.07)
**Religion**^**a**^		
Muslim	3828 (92.11)	2282 (59.61)
Non-Muslim	328 (7.89)	164 (49.99)
**Wealth index**^**c**^		
Poorest	679 (16.32)	504 (74.24)
Poorer	769 (18.49)	491 (63.90)
Middle	821 (19.74)	495 (60.36)
Richer	938 (22.55)	523 (55.74)
Richest	952 (22.90)	432 (45.41)
**Family size**^**a**^		
Small (≤ 4 members)	1768 (42.56)	1106 (62.56)
Large (> 4 members)	2387 (57.44)	1339 (56.10)
**Place of residence**^**d**^		
Urban	1275 (30.68)	696 (54.57)
Rural	2881 (69.32)	1750 (60.73)
**Division**^**e**^		
Barisal	209 (5.03)	135 (64.63)
Chattogram	814 (19.59)	440 (54.07)
Dhaka	1139 (27.41)	656 (57.57)
Khulna	391 (9.41)	241 (61.62)
Mymensingh	306 (7.37)	197 (64.39)
Rajshahi	520 (12.50)	364 (70.11)
Rangpur	433 (10.41)	290 (66.96)
Sylhet	344 (8.27)	122 (35.36)
**Community-level illiteracy**^**b**^		
< 25 (low)	966 (23.21)	495 (51.26)
25 to 50 (Moderate)	2396 (57.59)	1412 (58.95)
> 50 (high)	799 (19.20)	538 (67.36)
**Community-level poverty**^**c**^		
> 41.0 (High poverty)	1820 (43.77)	1185 (65.09)
16–41 (Moderate poverty)	1029 (24.75)	611 (59.39)
< = 15 (Low poverty)	560 (13.45)	255 (45.54)
Middle or rich community	749 (18.02)	395 (52.70)

^a^Estimates were adjusted or weighted by all women factor-total

^b^Estimates were adjusted or weighted by all women factor-education

^c^Estimates were adjusted or weighted by all women factor-wealth quintile

^d^Estimates were adjusted or weighted by all women factor-urban/rural

^e^Estimates were adjusted or weighted by all women factor-regional

^f^There were some missing values in husband’s education (*n* = 4019)

^g^Column percentage

^h^Row percentage

### Distribution of the prevalence of early marriage

The prevalence of early marriage was 58.85% (95%CI: 57.21–60.47). However, the reported prevalence was not consistent across women’s individual-, household- and community-level factors (Table 1). The prevalence of early marriage was substantially higher among women who completed up to secondary or less education (72–75%) and among Muslim women (60%). Around three-quarters of the early married women reported their husbands did not receive a formal education. With around 74% of early marriage, the prevalence was very high among women of poor households and it declined to 45% among women from households of the richest wealth quintiles. Early marriage was higher among rural women and those who live in the Rangpur, Mymensingh, Khulna and Barishal divisions. The prevalence was also found higher in communities with a high and moderate-level of poverty (Table 1).

### Cluster-wise variation of early marriage in Bangladesh

**Fig. 1:**
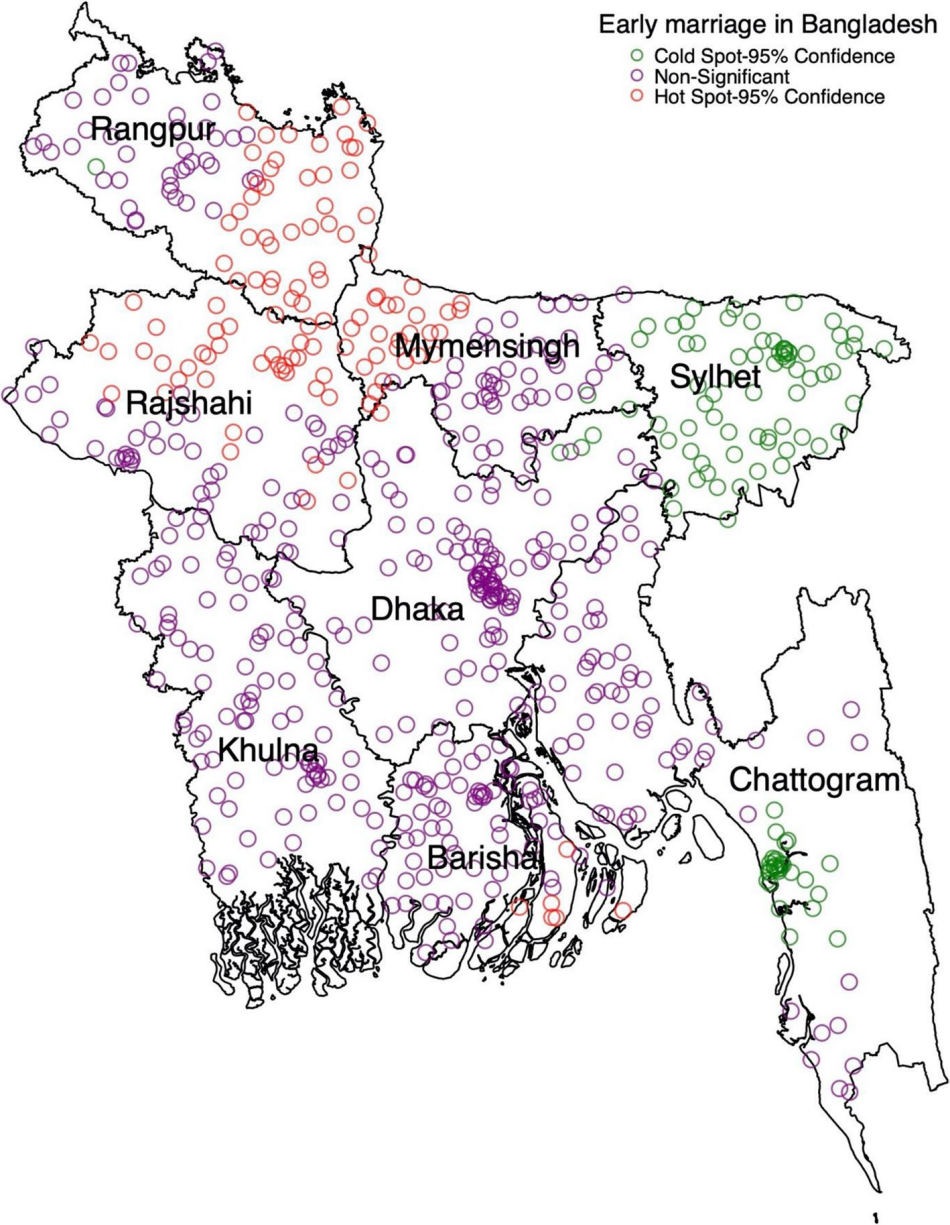
Hot-spot and cold-spots of early marriage in Bangladesh

Global Moran’s I value (0.482, *p* < 0.001) indicates a significant positive autocorrelation, and the Getis-Ord General G statistics reveal the presence of high clustering of early marriage in Bangladesh (z-score = 6.82, *p* < 0.001). The hot spots are located mainly in the Rangpur, Mymensingh and Rajshahi divisions. The cold spots are located in Sylhet and Chattogram divisions (Fig. 1). We also reported difference in hot spot and cold spot across socio-economic level of the respondents (result not shown in the table or figure).

### Factors associated with early marriage in Bangladesh

The results of the Poisson regression model for the predictors of early marriage are presented in Table 2. In the final model, we found the likelihood of early marriage declined by 9% (aPR: 0.91; 95% CI: 0.83–0.99) and 55% (aPR: 0.45; 95% CI: 0.40, 0.52) among secondary and higher educated women, respectively, as compared to the women who received no formal education. The likelihood of early marriage also declined by around 6–14% among women whose husbands received primary to higher education as compared to the women whose husbands received no formal education. Early marriage was 11% lower (aPR: 0.89; 95% CI: 0.79, 0.99) among Muslim women than non-Muslim women. As compared to the women in the Sylhet division, the prevalence of early marriage was higher among women in the Rajshahi (aPR: 1.63; 95% CI: 1.45, 1.83), Rangpur (aPR: 1.59; 95% CI: 1.41, 1.78), Barishal (aPR: 1.54; 95% CI: 1.35, 1.74), Khulna (aPR: 1.52; 95% CI: 1.35, 1.73), Mymensingh (aPR: 1.46; 95% CI: 1.29, 1.64), Dhaka (aPR: 1.40; 95% CI: 1.23, 1.59) and Chattogram (aPR: 1.38; 95% CI: 1.22, 1.56) divisions. The prevalence of early marriage was also higher among women residing in the community with moderate (aPR: 1.17; 95% CI: 1.03, 1.32) to a higher level of poverty (aPR: 1.16; 95% CI: 1.04, 1.29) as compared to the women in the community with a lower level of poverty.

**Table 2:** Determinants (multilevel Poisson model) and fitted model of early marriage among the women 20–24 years in Bangladesh, 2017/18^a^

**Variables**	**Null model**	**Individual-level model**	**Household-level model**	**Community-level model**	**Final model**
		**aPR (95% CI)**	**aPR (95% CI)**	**aPR (95% CI)**	**aPR (95% CI)**
***Women’s education***					
No formal education (reference)		1.00			1.00
Primary		0.97 (0.88, 1.06)			0.93 (0.85, 1.02)
Secondary		0.97 (0.88, 1.06)			0.91 (0.83, 0.99)*
Higher		0.49 (0.42, 0.56)***			0.45 (0.40, 0.52)***
***Husbands’ education***					
No formal education (reference)		1.00			1.00
Primary		0.93 (0.88, 0.99)*			0.94 (0.89, 0.99)*
Secondary		0.89 (0.83, 0.95)***			0.92 (0.86, 0.98)*
Higher		0.85 (0.77, 0.95)**			0.86 (0.77, 0.97)*
**Media exposure**					
No (reference)		1.00			1.00
Yes		1.01 (0.96, 1.07)			1.02 (0.96, 1.07)
***Family size***					
Small (reference)			1.00		1.00
Large			0.91 (0.87, 0.95)***		0.97 (0.93, 1.00)
***Wealth index***					
Poorer (reference)			1.00		1.00
Poor			0.89 (0.83, 0.96)**		0.94 (0.87, 1.01)
Middle			0.88 (0.82, 0.94)***		1.00 (0.93, 1.08)
Rich			0.80 (0.74, 0.86)***		1.01 (0.93, 1.11)
Richer			0.72 (0.66, 0.78)***		1.06 (0.95, 1.18)
***Religion***					
Muslim (reference)			1.00		1.00
Non-Muslim			0.82 (0.73, 0.92)***		.89 (0.79, 0.99)*
***Place of residence***					
Urban (reference)				1.00	1.00
Rural				0.94 (0.88, 1.02)	.97 (0.90, 1.04)
***Region***					
Sylhet (reference)				1.00	1.00
Barishal				1.47 (1.29, 1.68)***	1.54 (1.35, 1.74)***
Chattogram				1.36 (1.20, 1.54)***	1.38 (1.22, 1.56)***
Dhaka				1.40 (1.22, 1.59)***	1.40 (1.23, 1.59)***
Khulna				1.45 (1.27, 1.65)***	1.52 (1.35, 1.73)***
Mymensingh				1.40 (1.24, 1.59(***	1.45 (1.29, 1.64)***
Rajshahi				1.58 (1.40, 1.78)***	1.63 (1.45, 1.83)***
Rangpur				1.48 (1.31, 1.67)***	1.59 (1.41, 1.78)***
***Community-level illiteracy***					
< 25 (Low) (reference)				1.00	1.00
25–50 (Moderate)				1.04 (0.96, 1.12)	0.97 (0.90, 1.04)
> 50 (High)				1.11 (1.01, 1.22)*	0.97 (0.89, 1.06)
***Community-level poverty***					
Middle or rich (reference)				1.00	1.00
<= 15 (Low poverty)				0.89 (0.78, 1.02)	0.94 (0.83, 1.07)
16–41 (Moderate poverty)				1.11 (1.00, 1.24)	1.16 (1.04, 1.29)**
> 41 (High poverty)				1.16 (1.02, 1.30)*	1.17 (1.03, 1.32)*
Constant	58.85 (0.829)***	74.30 (68.03, 81.14)***	74.15 (70.53, 77.96)***	38.61 (33.37, 44.66)***	51.91 (43.52, 61.93)***

The first model contains only individual-level determinants (women’s education, husbands’ education, media exposure)

The second model contains only family-level determinants (family size, religion, and wealth index)

The third model contains only community-level determinants (place of residence and division, community-level literacy, and poverty)

The final model includes both individual- and community-level determinants (women’s education, husbands’ education, media exposure, family size, religion, wealth index, place of residence, division, community-level literacy and poverty)

*PR* Prevalence rate, *CI* Confidence interval

The asterisk indicates the significance level * at *p* > 0.05, ** at *p* > 0.01 and *** at *p* > 0.001

^a^All the estimates were adjusted/weighted by all women factor considering complex survey design with linearized error variance

### Model selection

Table 3 presents the AIC, BIC and ICC values of the five models that were run separately for assessing the predictors of early marriage. The best model is one which has the lowest AIC, BIC, and ICC values. According to these markers, final model, the combined model, was the best. We found around 20% variation in early marriage across the clusters before adjusting for the individual-, household- and community-level factors. The variation was reduced to 7% after adjustments in the final model.

**Table 3:** Intra class correlation (ICC), variances for random intercepts, Akaike Information Criteria (AIC), and Bayesian Information Criteria (BIC) for early marriage, Bangladesh 2017–18

**Models**	**Prevalence of early marriage (ref: No)**			
***Statistics***	***Intra class coefficient***	***Variance of random intercept***	***Akaike Information Criteria***	***Bayesian Information Criteria***
Null model	0.20	1.38 (0.56)	9514.23	8247.12
Individual-level model	0.14	1.20 (0.30)	8925.36	7415.36
Household-level model	0.11	1.14 (0.28)	7256.32	7058.36
Community-level model	0.09	1.11 (0.26)	6125.36	6982.32
Final model	0.07	0.90 (0.21)	4561.36	4156.36

## Discussion

This study aimed to explore the hot spots and cold spots of early marriage and the predictors of early marriage in Bangladesh. We found hot spots of early marriage were located mostly in the Mymensingh, Rangpur and Rajshahi divisions and cold spots were located mostly in the Sylhet division. The likelihood of early marriage declined with increasing levels of education of women as well as their husbands. The prevalence of early marriage was higher among Muslim than non-Muslim women. There were higher likelihoods of early marriage among women in all except the Sylhet division, and among women who resided in the community with moderate to higher poverty. We generated these results by using advanced statistical modelling in the most recent nationally representative survey data. Therefore, the findings are robust and should be used to refer to the present situation of Bangladesh as well as develop relevant policies and programs.

We found around 59% occurrence of early marriage. The hot spots of early marriage in Bangladesh are mainly located in parts of the Mymensingh, Rajshahi, and Rangpur divisions. This finding is novel and so we could not compare this finding with that in the existing literature. However, previous studies reported a higher prevalence of early marriage in the Rajshahi and Rangpur divisions [50]. This variation could be due to differences in socio-cultural factors such as area-level norms and traditions, education levels [4, 41, 50], employment and economic conditions [4, 41, 50, 69]. Also, the existing misconception and prejudice about early marriage vary across regions [70].

Administrative failure to reduce early marriage could be another important reason for the divisional variation. Bangladesh has four layers of administrative structure (division, district, upazila, and union *parishad*), in which union parishad and upazila are at the bottom. Although policies and programs are taken mainly on the divisional levels, upazila and union parishad administrations play critical roles in preventing early marriage. They operate awareness-building programs and impose relevant laws at the root level to prevent early marriage [71]. It is also common in Bangladesh that upazila and union parishad administrations stop early marriages on the day of occurrence by reaching the victims’ homes. They usually do so upon receiving information through their official channels (e.g., hotlines) as well as other sources [71, 72]. Some upazila and union parishads have been able to declare their jurisdictions free from child marriage [72]. It is likely that inadequate efforts by upazila and union parishads are made in areas identified as hot spots. Comparing administration-level activities in early marriage prevention in hot spots and cold spots may provide useful information about future approaches to intervention. It is also crucial to develop local-level policies and programs instead of the current top-down approach.

An important observation of this study was that the prevalence of early marriage decreases with increasing education levels. This observation is consistent both for women and their husbands. Each additional year of education may delay marriage [73]. This observation is similar to the existing literature [28, 30, 44, 48, 74]. Educated people are usually aware of the adverse effects of early marriage and may have the ability to fight against societal norms, culture and religious misunderstandings. Education may also give girls and boys decision-making abilities [14, 41, 56, 75].

In this study, we found a higher occurrence of early marriage among Muslim women than their non-Muslim counterparts which is consistent with previous studies in Bangladesh [10, 34, 42, 43, 56, 76]. The reason should be traditional norms of giving marriage early, mostly soon after the girls experience menstruation, that comes from misinterpretations of the religious doctrines [7, 14, 32, 34, 37, 38, 41, 43, 44, 46–49]. Also, preference for finding suitable brides/grooms within the same caste still prevails among Hindu communities, which constitute the lion part of the non-Muslim people in Bangladesh. This caste system together with a relatively small population makes the size of the eligible young people’s pool relatively smaller which subsequently reduces the chance of child marriage among the Hindu communities.

With growing access to the internet and social media, Bangladeshi communities are heavily influenced by western cultures. As such, a significant change has been noticed among school-going girls, including pre-marital sexual relationships [77–79]. Sexual relationships outside marriage are strictly prohibited in Islam and may put a girl in an extremely vulnerable situation [80]. If a young female is involved in an affair or is exposed to premarital sex, parents have no choice but to favour an immediate marriage, even if she is under-aged. This cultural change and fear about their children’s’ pre-marital relationship may motivate some parents to marry off their girls even if they are below 18.

As found in this study, community-level poverty is a cause of early marriage. We could not make any direct comparison of this finding because of the lack of relevant studies. There are several possible reasons for such associations. Poverty and education are intricately connected. A community where most people are relatively poor may not be aware of the adverse effects of early marriage. Early marriage may be a common social norm. Even if a small subset of parents is unwilling to early marriage, they may prefer to follow the community norms due to societal pressure. Moreover, in poorer households, parents are usually less interested to invest for their daughters as much they like to do for their sons. The reason could be community-level perception and tradition that girls are ultimately for grooms’ families. Sons stay with parents lifelong. Consequently, some parents may consider investing in their daughters’ education has little value and prefer to marry off as early as possible. This may also save expenditures associated with rearing, educating, and supporting until their marriages. Besides, girls’ marriage at a later age may necessitate providing dowry during marriage, although by law, it is completely prohibited in Bangladesh [7, 48, 81].

### Strengths and limitations

This study has several strengths. We demonstrated the variation in early marriage at the small geographic clusters using nationally representative data and robust statistical procedures. We also have identified influential factors of early marriage at the individual-, family- and community-level and developed the most parsimonious model that included all three layers of factors. Multilevel Poisson regression added further strength for hierarchical data when the prevalence of the outcome variable is relatively high. Besides, a large sample of data reduces variance and help predicts values that are close to the population parameters. The cross-sectional nature of the study design, however, may impact causality. Also, data on the unrevealed exposures that maybe intricately connected to early marriage, such as parental characteristics, familial and social circumstances, and psychosocial incentives for early marriage were not available.

## Conclusion

Our study found Mymensingh, Rangpur and Rajshahi divisions and some parts of the Barishal division had relatively high prevalence of early marriage. Women and their husbands who received relatively low levels of education, from Muslim families and moderate or high community-level poverty were more likely than their counterparts to experience early marriage. The findings are likely to be robust and suitable for informing evidence-based policies and programmes. Promoting girls’ and boys’ education, mandatory registration of all marriages, strict enforcement of the legal age of marriage, raising community awareness of the adverse effects of child marriage, and improving employment opportunities for women are some of the pragmatic programmes that can reduce the relatively high rate of early marriage in Bangladesh.

## Data Availability

The dataset can download after registering with the MEASURE DHS. Necessary information is available at: http://dhsprogram.com/data/Using-DataSets-for-Analysis.cfm.

## Abbreviations

LMICs: Low- and Middle-Income Countries
DHS: Demographic Health Survey
BDHS: Bangladesh Demographic Health Survey
AIC: Akaike Information Criteria
BIC: Bayesian Information Criteria
ICC: Intra-Class Correlation Coefficient
aPR: Adjusted Prevalence Ratio
CI: Confidence Interval
SDG: Sustainable Development Goal
NIPORT: National Institute of Population Research and Training
PSU: Primary Sampling Unit
GPS: Global Positioning System
